# Are there distinct patterns of mental and somatic symptoms in fibromyalgia? A pilot analysis.

**DOI:** 10.1192/j.eurpsy.2026.11478

**Published:** 2026-07-31

**Authors:** P. J. Turlińska, A. J. Krupa, M. Siwek

**Affiliations:** 1 Clinical Department of Adult Psychiatry, University Hospital; 2 Department of Affective Disorders, Jagiellonian University Medical College, Kraków, Poland

## Abstract

**Introduction:**

Fibromyalgia (FM) is a chronic condition characterized by widespread pain, fatigue, stiffness, as well as affective and sleep disturbances. (Wolfe et al., 2016) Increasing evidence highlights that FM represents a highly heterogeneous disorder, encompassing diverse constellations of somatic, affective, and psychosomatic symptoms rather than a uniform clinical entity.The current knowledge on fibromyalgia etiopathogenesis is incomplete and does not warrant a comprehensive description. Psychiatric comorbidities most notably depression and anxiety are closely related to the severity of FM symptoms. Our previous findings indicate that certain psychopathological dimensions, including depression, anxiety, anhedonia, chronobiological preferences, circadian rhythm disruptions, and specific psychological traits, are linked to poor pharmacological treatment response. (Krupa, Chrobak, et al., 2023; Krupa et al., 2025; Krupa, Korkosz, et al., 2023)

**Objectives:**

The goal of the study was to identify distinct symptomatologic clusters of patients diagnosed with FM.

**Methods:**

The cluster analysis was applied to the data from 77 patients with fibromyalgia including six key symptomatic variables: pain intensity, fatigue, anxiety, depression, cognitive difficulties, and morning well-being as assessed on a 11-point Likert scale (0-10).The clinical presentation was assessed by Fibromyalgia Impact Questionnaire (FIQ).

**Results:**

The mean FIQ score in the studied population was 54.46 points, indicating a moderate to severe level of symptom severity within the group. Clustering analysis revealed distinct subgroups of patients that differ significantly in the severity of physical and psychological symptoms. **1: Predominantly Physical (~25% of the group):** characterized by a predominance of somatic symptoms with moderate pain (mean 6.4) and high fatigue (mean 7.9), but low levels of anxiety (mean 1.9) and depression (mean 2.8). and mean FIQ score is 53.3, classifying this group as moderately severe. **2: Full-range (~56% of the group):** constituting the largest subgroup of patients presenting both mental and somatic symptoms. It is characterized by moderate pain (mean 6.5) and fatigue (mean 7.9), but high anxiety (mean 6.1) and depression (mean 6.7). This group demonstrates the the highest severity of illness and its’ impact on patients functioning (mean FIQ score of 65. **Mild (~19% of the group):** exhibiting minimal symptoms in all studied domains: low pain (mean 3.3), fatigue (mean 2.9), anxiety (mean 2.6), and depression (mean 2.6), with the mean FIQ score is 25.7 points.

**Image 1:**
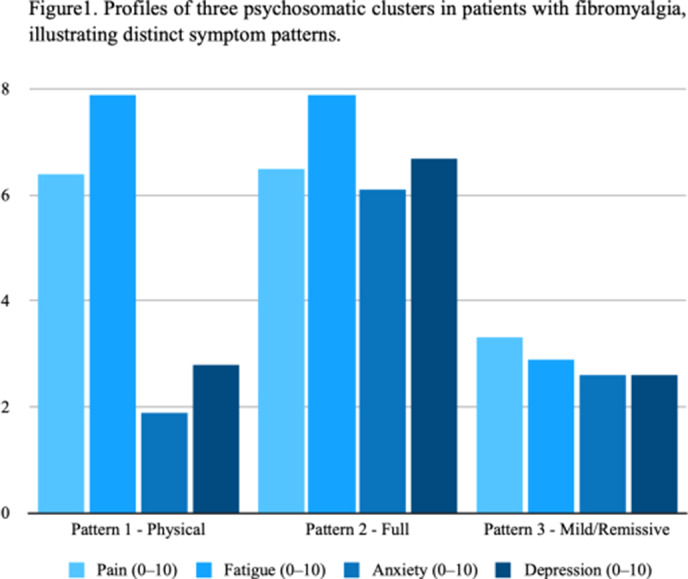
Image 1: Long description.

**Conclusions:**

The results show that distinct symptomatological patterns can be identified in fibromyalgia patients . These data could aid clinical practice however, they need to be verified in a larger group of patients.

**Disclosure of Interest:**

None Declared

## Long descriptions

### Image 1: Long description

The Y-axis represents symptom severity on a scale from 0 to 8. The X-axis categorizes three groups: Pattern 1 - Physical, Pattern 2 - Full, and Pattern 3 - Mild/Remissive. A legend at the bottom identifies four symptoms: Pain (lightest blue), Fatigue (medium-light blue), Anxiety (medium-dark blue), and Depression (darkest blue), all measured on a 0-10 scale.

* Pattern 1 - Physical: Pain and Fatigue are high, both near 6.5 and 8 respectively. Anxiety and Depression are significantly lower, both below 3.

* Pattern 2 - Full: All four symptoms are high. Pain is near 6.5, Fatigue is near 8, Anxiety is near 6, and Depression is near 7.

* Pattern 3 - Mild/Remissive: All symptoms are low, ranging between 2.5 and 3.5. Pain is the highest in this group at approximately 3.5, while the other three symptoms are clustered around 2.5.

Navigate back to Image 1:.

